# T helper cells in synovial fluid of patients with rheumatoid arthritis primarily have a Th1 and a CXCR3^+^Th2 phenotype

**DOI:** 10.1186/s13075-020-02349-y

**Published:** 2020-10-16

**Authors:** Jonathan Aldridge, Anna-Karin H. Ekwall, Linda Mark, Beatrice Bergström, Kerstin Andersson, Inger Gjertsson, Anna-Carin Lundell, Anna Rudin

**Affiliations:** Department of Rheumatology and Inflammation Research, Institute of Medicine, Sahlgrenska Academy, University of Gothenburg, Box 480, 405 30 Gothenburg, Sweden

**Keywords:** Rheumatoid arthritis, Th1, Th2, TPh, TFh, Fibroblast-like synoviocytes, Cytokines, Chemokines

## Abstract

**Background:**

The majority of CD4^+^ T helper (Th) cells found in the synovial fluid (SF) of patients with rheumatoid arthritis (RA) express CXCR3, a receptor associated with Th1 cells. In blood, subsets of Th2 and Th17 cells also express CXCR3, but it is unknown if these cells are present in RA SF or how cytokines from these subsets affect cytokine/chemokine secretion by fibroblast-like synoviocytes (FLS) from patients with RA.

**Methods:**

We examined the proportions of Th1, Th2, CXCR3^+^Th2, Th17, CXCR3^+^Th17, Th1Th17, peripheral T helper (TPh) and T follicular helper (TFh) cells in paired SF and blood, as well as the phenotype of TPh and TFh cells in RA SF (*n* = 8), by the use of flow cytometry. We also examined the cytokine/chemokine profile in paired SF and plasma (*n* = 8) and in culture supernatants of FLS from patients with chronic RA (*n* = 7) stimulated with Th-associated cytokines, by the use of cytometric bead arrays and ELISA. Cytokine receptor expression in FLS (*n* = 3) were assessed by the use of RNA sequencing and qPCR.

**Results:**

The proportions of Th1 and CXCR3^+^Th2 cells were higher in SF than in blood (*P* < 0.05). TPh and PD-1^high^TFh in RA SF were primarily of a Th1 and a CXCR3^+^Th2 phenotype. Moreover, the levels of CXCL9, CXCL10, CCL20, CCL2, CXCL8, IL-6 and IL-10 were higher in SF than in plasma (*P* < 0.05). Lastly, IL-4, IL-13 and IL-17A induced RA FLS to secrete proinflammatory IL-6, CCL2, CXCL1 and CXCL8, while IFNγ mainly induced CXCL10.

**Conclusion:**

These findings indicate that not only Th1 but also CXCR3^+^Th2 cells may have a pathogenic role in RA synovial inflammation.

## Background

Rheumatoid arthritis (RA) is a highly heterogeneous systemic chronic autoimmune disease that affects approximately 0.5–1% of individuals across different populations worldwide [1]. RA is characterised by inflammation of the synovial membrane and destruction of the cartilage and adjacent bone in the joints. In RA, the pattern of immune cell infiltration differs between individuals and the synovial inflammation can be subdivided into different pathotypes. Approximately half of the patients display organised B and T cell infiltrates in the synovial tissue that forms ectopic lymphoid-like structures (lympho-myeloid pathotype) [2]. Other patients lack these organised structures and have a more diffuse infiltration of macrophages and monocytes (diffuse-myeloid pathotype), or lack the infiltration of immune cells (pauci-immune pathotype) [2]. Genetic association studies specifically implicate CD4^+^ T cells as key actors in the induction of RA [3]. The classical CD4^+^ T cell subsets T helper 1 (Th1) and Th17 have been the focus of T cell research in RA [4], while the role of Th2 and non-classical Th subsets has been less well studied. Indeed, the majority of Th cells found in the SF of patients with RA express the chemokine receptor CXCR3 [5], a receptor conventionally used to categorise Th1 cells. However, we and others have shown that subsets of circulating Th2 and Th17 cells in both healthy individuals and RA patients also express CXCR3 [6, 7]. When stimulated in vitro, these non-classical CXCR3^+^Th2 and CXCR3^+^Th17 cells produce IFNγ as well as their associated cytokines IL-4 and IL-17A, respectively [7]. However, it is unknown if CXCR3^+^Th2 and CXCR3^+^Th17 cells are part of the population of CXCR3^+^ Th cells found in RA synovial fluid (SF).

Recently, a novel population of CXCR5^neg^PD-1^high^ Th cells named peripheral T helper cells (TPh) has been described in RA. This subset is expanded in RA SF and tissue and shares the B cell supporting characteristics (e.g. expression of PD-1, ICOS and IL-21) of T follicular helper cells (TFh) [8]. It is unknown if TPh cell can be subcategorised into Th1, Th2 and Th17 phenotypes similarly to circulating TFh cells [9].

The role of fibroblast-like synoviocytes (FLS) as active mediators of inflammation in the RA synovium through production of cytokines and chemokines has been well established [10, 11]. Th1- and Th17-associated cytokines IFNγ and IL-17A are known to promote cytokine and chemokine production by RA FLS. However, individuals who later develop RA present with elevated levels of multiple Th-associated cytokines in both SF and blood before RA diagnosis, and different Th subsets may be involved at different developmental stages of RA [12, 13]. In untreated early RA (ueRA) patients, recent findings point to activated Th1 and Th2 pathways in joints with a high level of lymphocyte infiltration [2]. We have reported that the proportions of Th2 cells are elevated in blood from ueRA patients compared to healthy controls [7] and that proportions of circulating Th2 cells correlate with disease activity in male patients with ueRA [14]. Furthermore, elevated levels of IL-13, IL-2, IL-15 and IL-4 in the SF most strongly distinguished early RA patients from other forms of early arthritis [12]. Despite these findings, the effects of IL-4 and IL-13 on the production of cytokines and chemokines by FLS from patients with RA have not been evaluated.

To address these gaps in knowledge, we here examined the distribution of classical and non-classical Th subsets in paired SF and blood from patients with RA. We also subcategorised TPh and TFh cells from RA SF into four Th phenotypes based on their expression of CCR4, CCR6 and CXCR3. Additionally, we examined the effects of Th1-, Th2- and Th17-associated cytokines on the cytokine/chemokine secretion profile of FLS from patients with RA. We found a higher proportion of not only Th1 cells but also CXCR3^+^Th2 cells in RA SF compared to blood. The majority of TPh and PD-1^high^TFh in RA SF also expressed CXCR3 and were of a Th1 or a CXCR3^+^Th2 phenotype. Moreover, we found that not only IL-17 and IFNγ, but also IL-4 and IL-13 induced RA FLS to secrete proinflammatory cytokines/chemokines, which were also found at higher levels in RA SF compared to plasma.

## Methods

### Blood, synovial fluid and tissue samples

Blood and SF were collected from patients diagnosed with RA (*n* = 8) according to either the American College of Rheumatology (ACR) 1987 or the European League Against Rheumatism (EULAR) 2010 classification criteria at the Rheumatology Clinic, Sahlgrenska University Hospital, Gothenburg, Sweden [15, 16]. Key inclusion criteria were clinically active RA, ≥ 18 years of age and ≥ one swollen joint. Patients with other arthritides were excluded. Patient characteristics are shown in Table 1.

**Table 1:** Patient characteristics

Donation	Blood and synovial fluid (*n* = 8)	Synovial tissue (*n* = 9)
Age, years^a^	57 (30–72)	62 (27–70)
Disease duration, years^a^	6.5 (< 1–13)	10.5 (1–22)
Female sex, *n* (%)	6 (75)	8 (89)
ACPA^+^ and/or RF+, *n* (%)	6 (75)	5 (56)
CRP, mg/L^a^	25 (1–54)	N/A
ESR, mm/h^a^	51 (11–64)	N/A
SJC28^a^	1.5 (1–5)	N/A
TJC28^a^	1 (1–4)	N/A

*N/A* data at the time of donation is not available

^a^Median and range

Synovial tissue samples were collected from RA patients undergoing joint replacement surgery (*n* = 9). All RA patients fulfilled the ACR 1987 revised criteria [16]. Only patients with established RA, ≥ 18 years of age and in a state of low disease activity were included. Samples from patients with other arthritides were excluded. Patient characteristics are shown in Table 1. The study was performed in compliance with the declaration of Helsinki, the regional ethics committee of Gothenburg approved all procedures and all patients provided written informed consent.

### Isolation and expansion of fibroblast-like synoviocytes

Synovial tissue samples from each of the nine respective patients were dissected into 1–2-mm segments and transferred to a tube containing 5 ml of Dulbecco’s modified Eagle’s medium (DMEM) GlutaMAX™ (Life Technologies Inc., Carlsbad, CA) with 25 μg/ml of Liberase™ TM (Roche, Mannheim, Germany) and incubated for 60 min. Dissolved tissue was rinsed twice in phosphate-buffered saline (PBS; HyClone™, GE Healthcare, Chicago, IL) and transferred into a culture flask containing 5 ml of complete media, i.e. DMEM GlutaMAX™ supplemented with 10% heat-inactivated foetal bovine serum (FBS; Life Technologies Inc.) and 50 mg/ml of gentamicin (Sigma-Aldrich, St. Louis, MO). Cells (FLS) were incubated at 37 °C at 5% CO_2_ and cultured until passage 4 before use. The number of patient samples used for each analysis is specified in the respective method sections.

### Flow cytometry

**Fig. 1:**
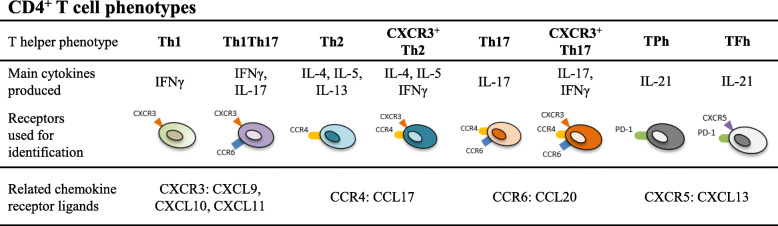
Summary of included T helper (Th) phenotypes, main cytokines produced [7, 8], receptors used for identification and related chemokine receptor ligands. TPh peripheral T helper cell, TFh T follicular helper cell

We identified the proportions of Th1, Th1Th17, Th2, CXCR3^+^Th2, Th17, CXCR3^+^Th17, TPh and TFh based on chemokine receptor expression in paired blood and SF from patients with active RA (*n* = 8) (summarised in Fig. 1) [7, 14]. Mononuclear cells were isolated by the use of Lymphoprep (Axis-Shield, Oslo, Norway) and freshly stained with fluorochrome-conjugated antibodies against CD4 (clone SK3, BD Bioscience, San Jose, CA), CD45RA (clone L48; BD Bioscience), CXCR3 (clone G025H7; BioLegend, San Diego, CA), CCR4 (clone L291H4, BioLegend), CCR6 (clone G034E3, BioLegend), CXCR5 (clone RF8B2; BD Bioscience) and PD-1 (clone EH12.2H7; BioLegend) followed by flow cytometry analysis. To exclude dead cells, 7-aminoactinomycin D (7-AAD, BD Bioscience) was used. Cells were acquired on a FACSCanto II (BD Bioscience) flow cytometer and FlowJo software (Tree Star Inc., Ashland, OR) was used to characterise Th subsets (gating strategy in Supplementary Figures 1 and 2).

Cultured primary FLS (passage 4–7) from patients with established RA (*n* = 5) were detached with 0.05% trypsin-EDTA (Life Technologies Inc.), washed in PBS and suspended in FACS buffer. Cells were stained with fluorochrome-conjugated antibodies against CD90 (clone 5E10, BioLegend), podoplanin (clone NZ-1.3; eBioscience, San Diego, CA) and CD55 (clone JS11, BioLegend). Viability dye eFluor® 506 (eBioscience) was used as viability stain. Cells were acquired using a BD FACSVerse (BD Bioscience) flow cytometer and analysed using FlowJo software (Tree Star Inc.). Fluorescence minus one was used to set gates for positive and negative populations.

### Stimulation of fibroblast-like synoviocytes

FLS from patients with established RA (*n* = 7) were cultured in U-bottomed 96-well plates (5000 cells/well) (TPP®, Trasadingen, Switzerland) in 85 μl of complete media with or without 10 ng/ml of IL-4 (Peprotech, London, UK), IL-13 (Peprotech), IL-17A (Peprotech), IFNγ (R&D Systems, Minneapolis, MN) or TNF (Life Technologies Inc.), respectively. Samples that contained added TNF were used as positive controls, and samples without an added cytokine were used as media controls. All stimulations were performed in duplicates. After 48 h, supernatants were collected and centrifuged for 5 min at 480*g* to remove cell debris and then stored at − 80 °C.

### RNA sequencing and qPCR

FLS from patients with established RA (*n* = 3) were serum starved (1% FBS) for 24 h and then lysed with RLT lysis buffer (Qiagen, Hilden, Germany), and total RNA was isolated using RNeasy® Micro kit (Qiagen). RNA sequencing was performed using the Nextseq500 platform, 2 × 75 read length and Nextseq500 Kit High Output V2 reagents. The library was prepared using TruSeq stranded Total RNA Sample preparation kit with Zero Gold according to the preparation guide (15031048 Rev. E). Data quality assessment was performed using FastQC (https://www.bioinformatics.babraham.ac.uk/projects/fastqc/). The Fastq files were filtered with Prinseq (version 0.20.3). Quality-filtered Fastq files were then mapped towards the human reference genome (hg19, UCSC assembly, February 2009) with STAR (version 2.5.2b). SAM tools (version 1.3.1) was used for alignment, sorting and indexing. Gene counts were calculated using Htseq (version 0.5.3p3).

For qPCR, total RNA was extracted from lysed RA FLS (*n* = 3) using a QIAcube with RNeasy® Micro kit (Qiagen). cDNA conversion was performed using a High Capacity cDNA Reverse Transcription kit with RNase inhibitor (Applied biosystems, Foster City, CA). The relative cDNA levels were determined by qPCR on a ViiA7 Real-Time PCR System (Thermo Fisher Scientific, Waltham, MA). Labelled primers for IL-4Rα (assay ID: Hs00166237_m1), IL-5Rα (Hs00602482_m1), IL-13Rα1 (Hs00609817_m1), IL-13Rα2 (Hs00152924_m1), IL-17RA (Hs01064648_m1), TNFRSF1A (Hs00533560_m1), TNFRSF1B (Hs00153550_m1) and IFNGR1 (Hs00988304_m1) along with GAPDH (Hs99999905_m1) (Taqman™; Applied biosystems) were used. Gene expression was normalised to GAPDH expression for each sample, respectively.

### Cytokine and chemokine analysis

The concentrations of cytokines/chemokines were measured in paired SF and plasma from patients with active RA (*n* = 8) and in culture supernatants from FLS derived from patients with established RA (*n* = 7). The cytokines/chemokines IL-2, IL-4, IL-5, IL-6, IL-9, IL-10, IL-13, IL-17A, IL-17F, IL-21, IL-22, IFNγ, TNF, CXCL1 (GROα), CXCL5 (ENA-78), CXCL8 (IL-8), CXCL9 (MIG), CXCL10 (IP-10), CXCL11 (I-TAC), CCL2 (MCP-1), CCL3 (MIP-1α), CCL4 (MIP-1β), CCL5 (RANTES), CCL11 (eotaxin), CCL17 (TARC) and CCL20 (MIP-3α) were analysed using three different bead-based immunoassays (LEGENDplex™; Human T Helper Cytokine Panel, Human Th17 Panel and Human Proinflammatory Chemokine Panel, BioLegend) in accordance with the manufacturer’s instructions. Samples were acquired on a FACSVerse (BD Bioscience) equipped with FACSuite software (BD Bioscience) and analysed by the use of FCAP Array software (Soft Flow Ltd., Pécs, Hungary). GM-CSF concentration in plasma and SF, and RANKL and GM-CSF concentrations in FLS supernatants were analysed using DuoSet® ELISA (R&D Systems) according to the manufacturer’s instructions.

### Statistical analysis

Non-parametric Wilcoxon matched-pairs signed rank test was used to compare proportions of Th subsets and cytokine/chemokine levels in paired samples of blood and SF (GraphPad Software, San Diego, CA). Orthogonal projection to latent structures discriminant analysis (OPLS-DA, SIMCA-P^+^ software; Umetrics, Umeå, Sweden) was performed in order to investigate if proportions of specific Th subsets associated with the SF or blood compartment in RA patients. Non-parametric Friedman test followed by Dunn’s multiple comparisons test was used to compare cytokine/chemokine levels between unstimulated (media control) and stimulated FLS (GraphPad Software). In all univariate analyses, a *P* value ≤ 0.05 was regarded as statistically significant.

## Results

### A substantial proportion of CXCR3^+^ T helper (Th) cells in RA synovial fluid are CXCR3^+^Th2 cells

**Fig. 2:**
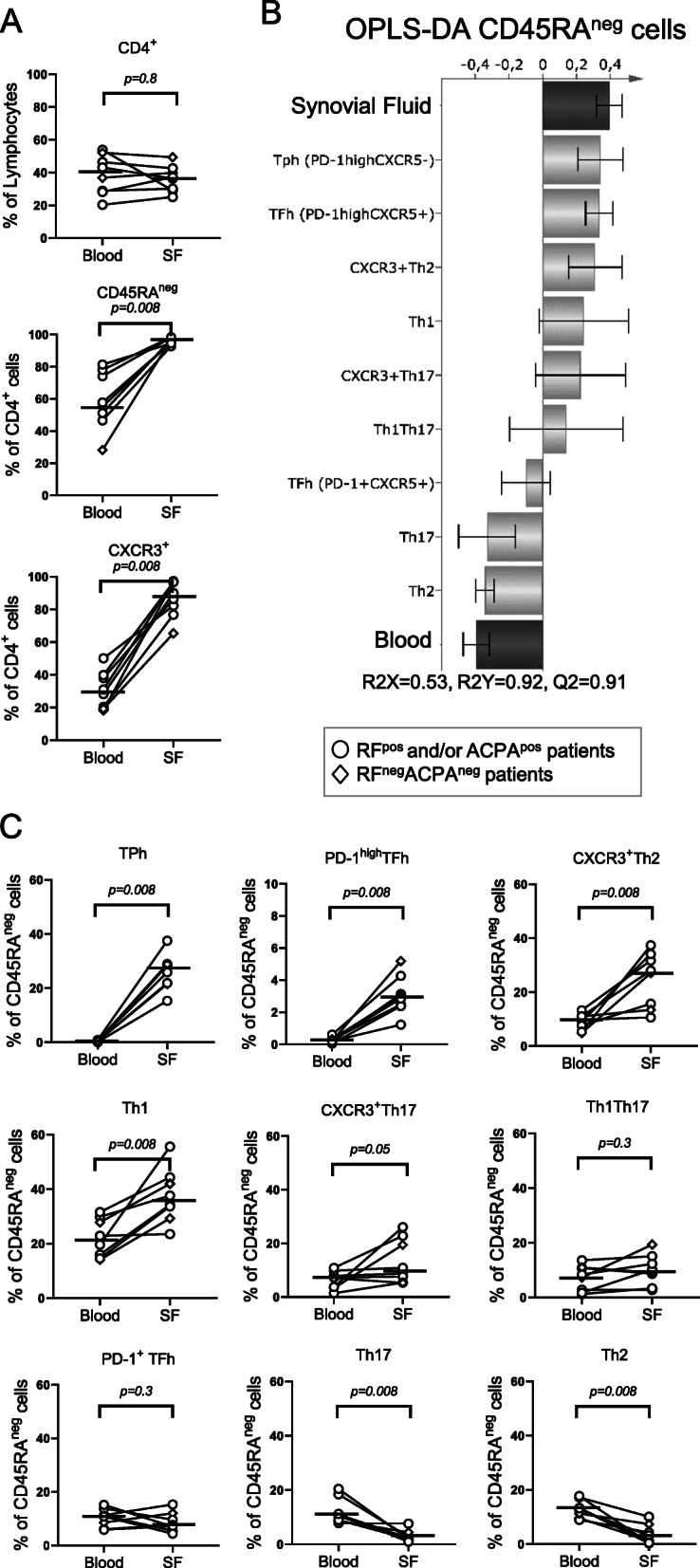
Distribution of memory T helper (Th) cell subsets in paired blood and synovial fluid (SF) from patients with rheumatoid arthritis (RA). **a** Comparison of the proportions of total CD4^+^ lymphocytes, CD45RA^neg^CD4^+^ memory T cells and CXCR3^+^CD4^+^ T cells in blood and SF of patients with RA (*n* = 8). **b** OPLS-DA column loading plots showing the association of CD45RA^neg^ Th subset proportions (X-variables) to either SF or blood (Y-variables). **c** Comparison of the proportions of TPh, PD-1^high^TFh, CXCR3^+^Th2, Th1, CXCR3^+^Th17, Th1Th17, PD-1^+^ TFh, Th17 and Th2 subsets of CD45RA^neg^CD4^+^ lymphocytes in blood and SF (gating strategy shown in Supplementary Figure 1). Horizontal bars indicate median. *P* value denotes the statistical significance of the difference between T cell proportions in blood and SF (Wilcoxon matched-pairs signed rank)

Several CD4^+^ Th subsets and their associated cytokines have been linked to RA disease pathogenesis. Most studies have focused on the Th1 and Th17 subsets, while Th2 cells as well as non-classical CXCR3^+^Th2 and CXCR3^+^Th17 cells have been less well studied. Therefore, Th subset proportions were examined in samples of paired SF and blood by the use of a comprehensive panel of Th subsets, as previously described [7, 14]. CD4^+^ cells were categorised into Th1, Th2, Th17, Th1Th17 and non-classical CXCR3^+^Th2 and CXCR3^+^Th17 subsets by their expression of CCR6, CCR4 and CXCR3 (gating strategy for blood and SF in Supplementary Figure 1A-B, respectively), and into TFh and TPh subsets by their expression of CXCR5 and PD-1 (gating strategy in Supplementary Figure 2). The proportion of CD4^+^ Th cells among lymphocytes did not differ between blood and SF (Fig. 2a), but as expected, the proportions of memory Th cells and CXCR3^+^ Th cells were significantly higher in SF than in blood (Fig. 2a). We then investigated the association of specific T cell subsets to either the SF or blood compartment by OPLS-DA. The proportions of TPh, PD-1^high^TFh, CXCR3^+^Th2 and Th1 cells associated positively with the SF compartment (Fig. 2b) and were significantly higher in SF than in blood (Fig. 2c). The CXCR3^+^Th17, Th1Th17 and PD-1^+^ TFh cell subsets were present in both SF and blood, but the proportions of these cells did not differ significantly between the two compartments. In contrast, the proportions of Th2 and Th17 cells associated positively with the blood compartment (Fig. 2b) and were significantly lower in SF compared to blood (Fig. 2c). There were no clear differences in Th subset distribution in RA SF and blood among RF^+^ and/or ACPA^+^ patients and RF^neg^ACPA^neg^ patients. Thus, in addition to confirming the association of the TPh and Th1 subsets with RA SF, our results show that a substantial proportion of CXCR3^+^ Th cells in RA SF are non-classical Th cells and that proportions of CXCR3^+^Th2 and PD-1^high^TFh cells were higher in RA SF compared to paired blood.

### The majority of SF TPh and PD-1^high^TFh cells are of a Th1 or CXCR3^+^Th2 phenotype in RA

**Fig. 3:**
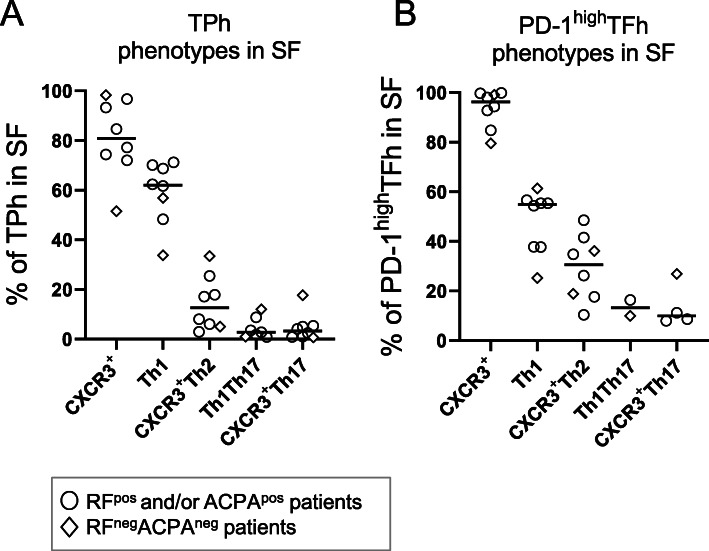
CXCR3^+^ expression and Th phenotypes of peripheral T helper (TPh) and PD-1^high^ T follicular helper (TFh) cells in synovial fluid (SF) from patients with rheumatoid arthritis (RA). The proportions of TPh (**a**) and PD-1^high^TFh (**b**) cells in RA SF that express CXCR3, and the proportions that express a Th1, CXCR3^+^Th2, Th1Th17 or CXCR3^+^Th17 phenotype as determined by the expression of chemokine receptors CCR6, CCR4 and CXCR3 (gating strategy shown in Supplementary Figure 2). Horizontal bars indicate the median

As the proportions of both TPh and PD-1^high^TFh were higher in RA SF than in blood, we subcategorised these subsets based on their expression of CCR6, CCR4 and CXCR3 (gating strategy shown in Supplementary Figure 2). The majority of TPh and PD-1^high^TFh in RA SF expressed CXCR3 and both subsets primarily consisted of cells with a Th1 or CXCR3^+^Th2 phenotype (Fig. 3a, b). Only a small fraction of TPh and PD-1^high^TFh cells in RA SF had a CXCR3^+^Th17 or Th1Th17 phenotype (Fig. 3a, b). The number of cells categorised as TPh and PD-1^high^TFh in blood samples was too few to subcategorise (< 50 cells/subgate). Thus, the proportion of Th phenotypes within TPh and PD-1^high^TFh cells reflects the CD4^+^ T cell population in RA SF where the majority of cells are of a Th1 and CXCR3^+^Th2 phenotype.

### Differences in chemokine and cytokine levels in paired synovial fluid and plasma in RA

**Fig. 4:**
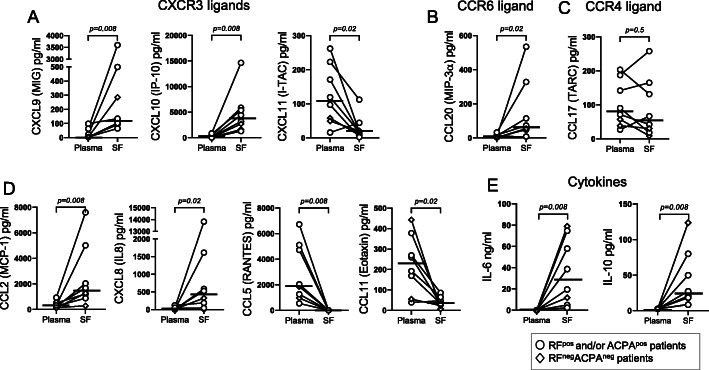
Chemokine and cytokine concentrations in paired plasma and synovial fluid (SF) from patients with rheumatoid arthritis (RA). **a** CXCL9 (MIG), CXCL10 (IP-10) and CXCL11 (I-TAC); **b** CCL20 (MIP-3α); **c** CCL17 (TARC); **d** CCL2 (MCP-1), CXCL8 (IL-8), CCL5 (RANTES) and CCL11 (eotaxin); **e** IL-6 and IL-10 concentrations in paired plasma and SF of patients with RA (*n* = 8) as measured by bead-based immunoassays. Horizontal bars indicate the median. *P* value denotes the statistical significance of the difference in concentration between plasma and SF (Wilcoxon matched-pairs signed rank test)

To investigate mechanisms for immune cell recruitment and cytokine signalling in the RA joint, we assayed paired SF and plasma samples for 27 chemokines and cytokines. Among the CXCR3 receptor ligands, the concentrations of CXCL9 and CXCL10 were significantly higher in SF than in plasma, while CXCL11 levels were lower in SF than in plasma (Fig. 4a). The level of the CCR6 ligand CCL20 was also detected at a higher concentration in SF than in plasma (Fig. 4b), while no such difference was observed for the CCR4 ligand CCL17 (Fig. 4c). Monocyte and neutrophil chemoattractants CCL2 and CXCL8, respectively, were both found at higher concentration in SF than in plasma (Fig. 4d). In contrast, CCL5 and eosinophil-recruiting chemokine CCL11 were found in higher concentrations in plasma than in SF (Fig. 4d). No differences were observed in the levels of CCL3, CCL4, CXCL1 and CXCL5 between plasma and SF (Supplementary Figure 3A). The concentrations of IL-6 and IL-10 were both higher in SF compared to plasma (Fig. 4e). The levels of the cytokines IL-4, IL-13, IL-9, IL-17A, IL-17F, IL-21, IFNγ and TNF were low in both plasma and SF and did not differ significantly (Supplementary Figure 3B). The concentrations of IL-2, IL-5, IL-22 and GM-CSF were below the detection limit of the assay (data not shown). There were no clear differences in cytokine/chemokine levels in RA SF and plasma among RF^+^ and/or ACPA^+^ patients and RF^neg^ACPA^neg^ patients. In conclusion, levels of chemokines that recruit in particular CXCR3^+^ T cells, together with proinflammatory CCL2, CXCL8 and IL-6, were higher in SF than in plasma.

### Th1-, Th2- and Th17-associated cytokines induce RA FLS to produce proinflammatory cytokines and chemokines

To evaluate the cytokine response by FLS in response to stimulation with various Th cell-associated cytokines, we first examined the surface expression of three fibroblast markers expressed by FLS in the RA synovium, CD90 (Thy-1), podoplanin and CD55 [17–19]. As shown in Supplementary Figure 4A-B, the majority of unstimulated RA FLS in culture expressed all three markers. Moreover, in a data set of gene expression from RNA sequencing of three primary unstimulated RA FLS samples, we found that the receptors of IL-4 (IL-4Rα) and IL-13 (IL-13Rα1 and IL-13Rα2), IL-17A (IL-17RA), TNF (TNFRSF1A and TNFRSF1B) and IFNγ (IFNGR1) were expressed by FLS, which was confirmed by qPCR (Supplementary Figure 4C).

**Fig. 5:**
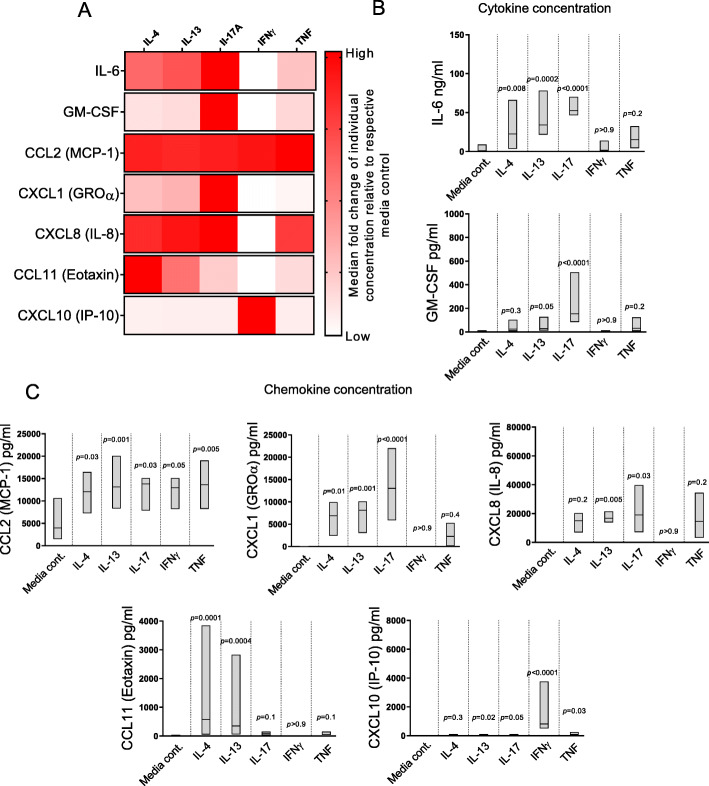
Fibroblast-like synoviocytes (FLS) from patients with rheumatoid arthritis (RA) produce proinflammatory cytokines and chemokines when stimulated with T helper-associated cytokines. **a** The median fold change in IL-6, GM-CSF, CCL2, CXCL1, CXCL8, CCL11 and CXCL10 concentration in supernatants of stimulated as compared to unstimulated FLS. **b** IL-6 and GM-CSF levels and **c** CCL2, CXCL1, IL-8, CCL11 and CXCL10 levels in cell culture supernatant of RA (*n* = 7) FLS stimulated with IL-4, IL-13, IL-17A, IFNγ or TNF as measured by bead-based immunoassays and ELISA. Lines within boxes indicate the median. *P* value denotes the statistical significance of the difference between respective cytokine stimulus and media controls (Friedman test followed by Dunn’s multiple comparisons test)

Next, we examined the cytokine/chemokine secretion profile of RA FLS cultured in the presence of IL-4, IL-13, IL-17A, IFNγ or TNF. As shown in Fig. 5a, b, stimulation with IL-4, IL-13 and IL-17A induced significantly higher levels of IL-6 by RA FLS compared to media control samples. IL-17A induced increased GM-CSF secretion from RA FLS, while IFNγ and TNF had no significant effect on either IL-6 or GM-CSF production. Levels of IL-2, IL-4, IL-5, IL-9, IL-10, IL-13, IL-17A, IL-17F, IL-21, IL-22, IFNγ, TNF and RANKL were below the detection limit of the assay or did not differ significantly between stimulated samples and media controls (data not shown). Regarding chemokine secretion, stimulation with IL-4, IL-13, IL-17A and TNF triggered a significant increase in CCL2 production by RA FLS compared to media controls (Fig. 5c). IL-4, IL-13 and IL-17A also significantly increased the levels of CXCL1 compared to media controls, while IL-13 and IL-17A increased the secreted levels of CXCL8 compared to media controls. Only IL-4 and IL-13 induced significantly higher levels of CCL11 by RA FLS compared to media controls. As expected, IFNγ was the most potent stimulus for CXCL10 secretion by RA FLS. Low levels of CXCL5, CXCL9, CXCL11, CCL3, CCL4, CCL5, CCL17 and CCL20 were detected in stimulated and media controls but no significant differences were found under the present experimental conditions (data not shown). In conclusion, these findings show that both Th2- and Th17-associated cytokines are able to induce the production of proinflammatory cytokines/chemokines IL-6, CCL2, CXCL1, CXCL8 and CCL11, by RA FLS.

## Discussion

The majority of CD4^+^ T cells found in the SF of RA patients express CXCR3, a characteristic of IFNγ-producing Th1 cells [5]. We have recently shown that subsets of circulating Th2 and Th17 cells also express CXCR3 [7], although it is not known if these cells are found in SF of RA patients. Additionally, it is not known how the TPh cells that are expanded in RA SF can be subcategorised in this respect. Lastly, in contrast to IFNγ, IL-17 and TNF, the effects of Th2-associated cytokines IL-4 and IL-13 on FLS secretion of cytokines/chemokines have not been studied. We here report that patients with RA have increased proportions of both Th1 and non-classical CXCR3^+^Th2 cells in SF compared to blood and that the majority of TPh and PD-1^high^TFh cells in RA SF are of a Th1 or CXCR3^+^Th2 phenotype. Furthermore, we report that Th2- and Th17-associated cytokines trigger the secretion of proinflammatory molecules such as IL-6, CCL2 and CXCL8, which were also present at a higher concentration in RA SF compared to plasma.

We have previously shown that untreated early RA (ueRA) patients have higher proportions of Th2 cells in the blood compared to healthy controls (HC), while the proportion of Th1 cells tended to be lower in ueRA patients than in HC [7]. In this study on clinical samples from patients with active RA, we find that the proportions of Th2 cells are lower in SF than in blood, while the proportions of CXCR3^+^Th2 and Th1 cells are significantly higher in SF. The lower proportion of Th1 cells in circulation of ueRA patients compared to HC may therefore be explained by a migration of the Th1 population from blood to SF. In established RA, others have found that approximately 90% of all CD4^+^ T cells in SF express CXCR3, although all of these cells were assumed to be Th1 cells in this study [5]. In the present study, we show that up to 37% of the CXCR3^+^ memory Th cells in RA SF are CXCR3^+^Th2 cells. In line with this, it was recently published that RA synovial tissue with a lympho-myeloid pathotype shows enrichment in both Th1 and Th2 activation pathways [2]. Thus, our findings indicate that both Th1 and CXCR3^+^Th2 cells may be important in the pathogenesis of RA joint inflammation.

TPh cells share characteristics of TFh cells and are able to promote plasma cell development in vitro [8]. TPh differ from TFh in that they lack CXCR5 expression and instead express the chemokine receptor CCR2 [8]. In line with previous findings [8], we find higher proportions of TPh cells in RA SF compared to blood, and we also find higher concentrations of the CCR2 ligand CCL2 in SF than in plasma. In contrast to the previous study [8], we also find a significantly higher proportion of PD-1^high^TFh cells in SF compared to blood. Circulating TFh from healthy individuals can be subcategorised into Th1, Th2 and Th17 phenotypes based on their expression of CXCR3 and CCR6 [9]. We here show that the majority of TPh and PD-1^high^TFh cells in RA SF also are of a Th1 or CXCR3^+^Th2 phenotype. Previous findings show that circulating TFh with a Th2 phenotype in healthy individuals can induce antibody production by naïve B cells in vitro, while TFh of a Th1 phenotype lacked this ability [9]. Further studies are needed to evaluate if this difference in B cell helping capacity exists in TPh and PD-1^high^TFh cells with a Th1 or a CXCR3^+^Th2 phenotype.

We have previously reported that plasma CXCL10 is associated with disease activity in early RA [20], and others have shown that serum CXCL10 may be used to predict treatment response in RA patients treated with anti-TNF therapy [21]. In contrast to CXCL10, no relation with disease activity has been shown for plasma CXCL9 [20]. We here show that the concentrations of CXCL9 and CXCL10 are significantly higher in SF than in plasma, but that IFNγ had a significant effect on the secretion of only CXCL10 by RA FLS. In line with these findings, others have shown that RA SF contains significantly higher levels of CXCL10 compared to SF from patients with OA or traumatic joint injury [5] and that deletion of either CXCL10 or its receptor CXCR3 significantly abrogates CD4^+^ T cell infiltration in murine arthritis models [22]. Thus, CXCL10 in RA SF may facilitate the recruitment of Th1 and CXCR3^+^Th2 cells. However, not all Th subsets that express CXCR3 were found at increased proportions in SF. The proportions of CXCR3^+^Th17 and Th1Th17 cells did not differ significantly between SF and blood despite a higher concentration of CCR6 ligand CCL20 in SF than in blood. Therefore, other factors may also be involved in the attraction of Th1 and CXCR3^+^Th2 cells to the RA joint. TPh cells express CCR2 [8], and the TPh population consists of cells with both a Th1 and a CXCR3^+^Th2 phenotype. Therefore, the combined expression of CXCR3 and CCR2 may contribute to the preferential attraction of Th1 and CXCR3^+^Th2 cells to the RA joint.

The importance of FLS as mediators of inflammation through the production of cytokines and chemokines in the RA synovium has been well established [10, 11]. However, in contrast to IFNγ, IL-17A and TNF, the effect of IL-4 and IL-13 on the secretion of cytokines and chemokines by RA FLS has not been studied. This is likely due to that IL-4 and IL-13 have been considered anti-inflammatory in RA [23]. In the present study, however, we found that IL-4 and IL-13 induced RA FLS to secrete multiple proinflammatory cytokines/chemokines, which were also found at higher concentrations in SF than in plasma. In fact, the levels of IL-6, CCL2 and CXCL8 secreted by RA FLS in vitro in response to IL-4 and IL-13 were comparable to those induced by IL-17. The higher levels of IL-6, CCL2 and CXCL8 in SF than in blood also suggest that they are produced locally in the joint. Indeed, recent findings show that FLS in the sub-lining layer of the RA synovium appear to be a primary source of IL-6 and CCL2 [10]. Moreover, it was shown that ueRA patients with a lympho-myeloid pathotype present with increased expression of the Th2 activation pathway [2]. These findings support that Th2-associated cytokines could be inducers of IL-6 and CCL2 production by FLS in the RA synovium also in vivo. The lympho-myeloid pathotype of RA frequently includes the formation of organised ectopic lymphoid-like structures (ELS), and the formation of ELS in Sjögren’s syndrome has been shown to be dependent on IL-13 signalling [24]. Specifically, IL-13 act synergistically with TNF to promote a pathogenic phenotype in salivary gland fibroblasts characterised by increased VCAM-1 expression [24]. IL-4 and IL-13 along with TNF have a similar effect on RA FLS, where these cytokines induce a sustained expression of VCAM-1 [25]. Collectively, these findings support a proinflammatory role of IL-4 and IL-13 in the RA synovium where they may facilitate the development of a lympho-myeloid pathotype.

The limitations of this study include the use of samples from a relatively low number of RA patients with a diverse treatment history and disease duration. This study intended to explore if non-classical Th subsets are present in RA SF, but additional studies are needed to verify these findings and to further investigate the specific role of CXCR3^+^Th2 cells in RA synovial inflammation.

## Conclusion

In conclusion, we demonstrate that T helper cells in RA SF consist mainly of Th1 cells and a population of CXCR3^+^Th2 cells not previously identified in RA SF. Thus, our findings indicate that both Th1 and CXCR3^+^Th2 cells may have a pathogenic role in RA synovial inflammation.

## Supplementary information

**Additional file 1: Supplementary Figure 1.** Gating strategy for T helper cells in paired blood and synovial fluid (SF) from patients with rheumatoid arthritis (RA). **Supplementary Figure 2.** Gating strategy for peripheral T helper (TPh) cells and T follicular helper (TFh) cells in synovial fluid (SF) from patients with rheumatoid arthritis (RA). **Supplementary Figure 3.** Chemokines and cytokines in plasma and synovial fluid (SF) of patients with rheumatoid arthritis (RA). **Supplementary Figure 4.** Expression of fibroblast markers and cytokine receptors by unstimulated cultured fibroblast-like synoviocytes (FLS) from patients with rheumatoid arthritis (RA).

## Data Availability

The datasets used during the present study are available from the corresponding author Jonathan Aldridge or Anna Rudin upon reasonable request.

## Abbreviations

ACR: American College of Rheumatology
ELISA: Enzyme-linked immunosorbent assay
EULAR: European League Against Rheumatism
FLS: Fibroblast-like synoviocytes
DMEM: Dulbecco’s modified Eagle’s medium
OPLS-DA: Orthogonal projection to latent structures discriminant analysis
PCR: Polymerase chain reaction
RA: Rheumatoid arthritis
SF: Synovial fluid
Th: T helper
TFh: T follicular helper
TPh: Peripheral T helper
TPM: Transcripts per kilobase million
ueRA: Untreated early rheumatoid arthritis
