# Supplementary material for: T helper cells in synovial fluid of patients with rheumatoid arthritis primarily have a Th1 and a CXCR3+Th2 phenotype
Source: Arthritis Res Ther. 2020 Oct 16;22:245. doi: 10.1186/s13075-020-02349-y (PMC7566124; doi:10.1186/s13075-020-02349-y)
Supplement: Supplementary file 1 — Additional file 1: Supplementary Figure 1. Gating strategy for T helper cells in paired blood and synovial fluid (SF) from patients with rheumatoid arthritis (RA). Supplementary Figure 2. Gating strategy for peripheral T helper (TPh) cells and T follicular helper (TFh) cells in synovial fluid (SF) from patients with rheumatoid arthritis (RA). Supplementary Figure 3. Chemokines and cytokines in plasma and synovial fluid (SF) of patients with rheumatoid arthritis (RA). Supplementary Figure 4. Expression of fibroblast markers and cytokine receptors by unstimulated cultured fibroblast-like synoviocytes (FLS) from patients with rheumatoid arthritis (RA). [file 13075_2020_2349_MOESM1_ESM.pptx]

## Slide 1
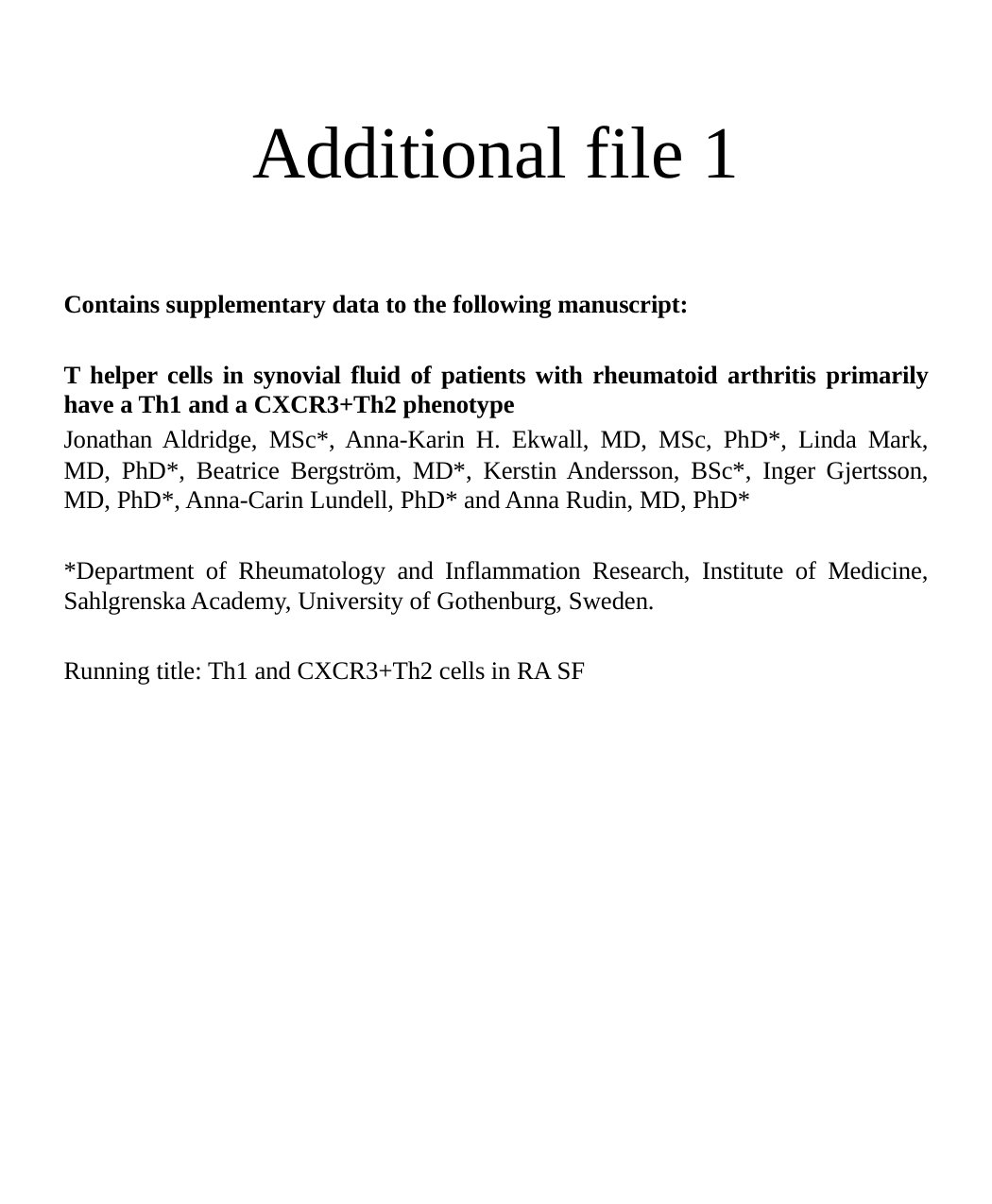

# Additional file 1
Contains supplementary data to the following manuscript:
T helper cells in synovial fluid of patients with rheumatoid arthritis primarily have a Th1 and a CXCR3+Th2 phenotype
Jonathan Aldridge, MSc*, Anna-Karin H. Ekwall, MD, MSc, PhD*, Linda Mark, MD, PhD*, Beatrice Bergström, MD*, Kerstin Andersson, BSc*, Inger Gjertsson, MD, PhD*, Anna-Carin Lundell, PhD* and Anna Rudin, MD, PhD*
*Department of Rheumatology and Inflammation Research, Institute of Medicine, Sahlgrenska Academy, University of Gothenburg, Sweden.
Running title: Th1 and CXCR3+Th2 cells in RA SF

## Slide 2
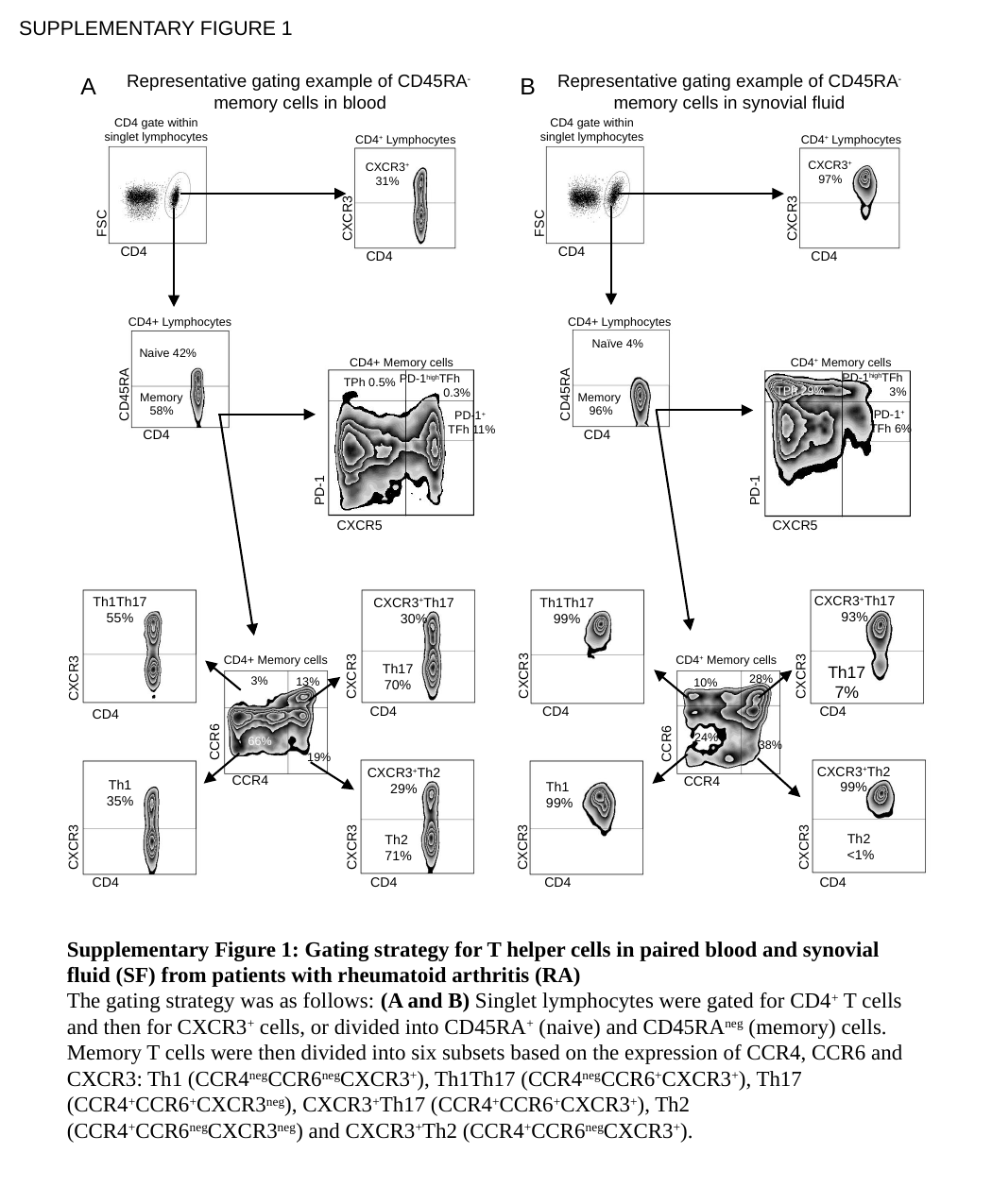

SUPPLEMENTARY FIGURE 1
Representative gating example of CD45RA- memory cells in synovial fluid
Representative gating example of CD45RA-
 memory cells in blood
A
B
CD4 gate within
singlet lymphocytes
FSC
CD4
CD4 gate within
singlet lymphocytes
CD4+ Lymphocytes
CD4+ Lymphocytes
CXCR3+
97%
CXCR3+
31%
FSC
CD4
CXCR3
CD4
CXCR3
CD4
CD4+ Lymphocytes
CD4+ Lymphocytes
CD45RA
CD4
Naïve 4%
Memory
 96%
CD45RA
CD4
Naive 42%
Memory
58%
CD4+ Memory cells
CD4+ Memory cells
PD-1highTFh
3%
PD-1highTFh 0.3%
TPh 0.5%
TPh 29%
PD-1+
TFh 6%
PD-1+
 TFh 11%
PD-1
PD-1
CXCR5
CXCR5
CXCR3+Th17
93%
Th1Th17
55%
CXCR3+Th17
30%
Th1Th17
99%
CXCR3
CD4
CXCR3
CD4
CXCR3
CD4
CXCR3
CD4
CD4+ Memory cells
CD4+ Memory cells
Th17
70%
Th17
7%
28%
3%
13%
10%
CCR6
CCR4
CCR6
CCR4
24%
66%
38%
19%
CXCR3+Th2
99%
CXCR3+Th2
29%
Th1
35%
Th1
 99%
CXCR3
CD4
CXCR3
CD4
CXCR3
CD4
CXCR3
CD4
Th2
 <1%
Th2
 71%
Supplementary Figure 1: Gating strategy for T helper cells in paired blood and synovial fluid (SF) from patients with rheumatoid arthritis (RA)
The gating strategy was as follows: (A and B) Singlet lymphocytes were gated for CD4+ T cells and then for CXCR3+ cells, or divided into CD45RA+ (naive) and CD45RAneg (memory) cells. Memory T cells were then divided into six subsets based on the expression of CCR4, CCR6 and CXCR3: Th1 (CCR4negCCR6negCXCR3+), Th1Th17 (CCR4negCCR6+CXCR3+), Th17 (CCR4+CCR6+CXCR3neg), CXCR3+Th17 (CCR4+CCR6+CXCR3+), Th2 (CCR4+CCR6negCXCR3neg) and CXCR3+Th2 (CCR4+CCR6negCXCR3+).

## Slide 3
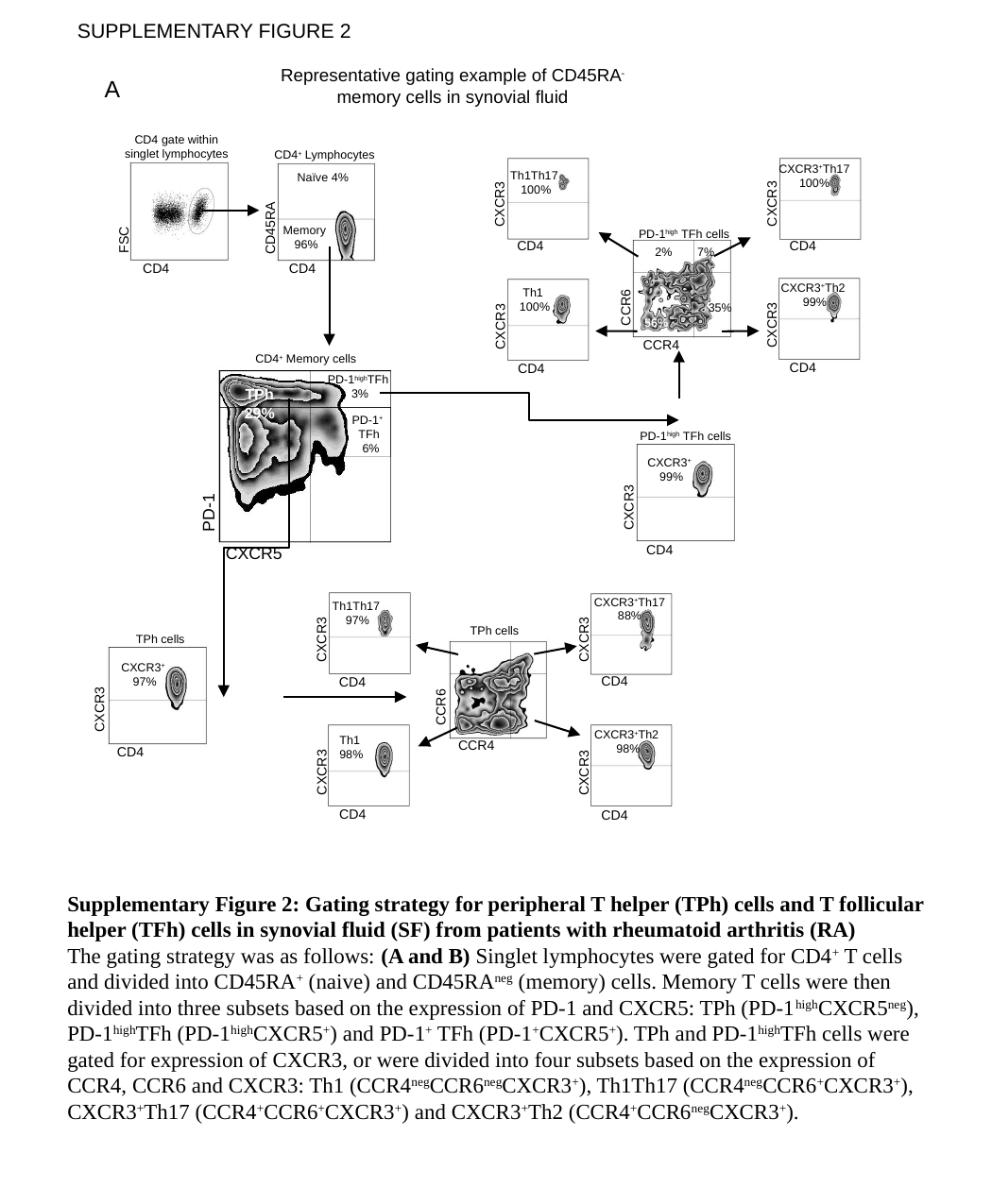

SUPPLEMENTARY FIGURE 2
Representative gating example of CD45RA- memory cells in synovial fluid
A
CD4 gate within
singlet lymphocytes
CD4+ Lymphocytes
CD45RA
CD4
Naïve 4%
Memory
 96%
CXCR3+Th17
100%
CXCR3
CD4
CXCR3
CD4
Th1Th17
 100%
FSC
CD4
PD-1high TFh cells
2%
7%
CCR6
CCR4
CXCR3+Th2
 99%
Th1
 100%
CXCR3
CD4
CXCR3
CD4
35%
56%
CD4+ Memory cells
PD-1highTFh
 3%
TPh 29%
PD-1+
TFh
 6%
PD-1high TFh cells
CXCR3+
 99%
CXCR3
CD4
PD-1
CXCR5
CXCR3+Th17
88%
Th1Th17
 97%
CXCR3
CD4
CXCR3
CD4
TPh cells
TPh cells
CXCR3+
 97%
CCR6
CCR4
CXCR3
CD4
CXCR3+Th2
 98%
Th1
 98%
CXCR3
CD4
CXCR3
CD4
Supplementary Figure 2: Gating strategy for peripheral T helper (TPh) cells and T follicular helper (TFh) cells in synovial fluid (SF) from patients with rheumatoid arthritis (RA)
The gating strategy was as follows: (A and B) Singlet lymphocytes were gated for CD4+ T cells and divided into CD45RA+ (naive) and CD45RAneg (memory) cells. Memory T cells were then divided into three subsets based on the expression of PD-1 and CXCR5: TPh (PD-1highCXCR5neg), PD‑1highTFh (PD-1highCXCR5+) and PD-1+ TFh (PD-1+CXCR5+). TPh and PD‑1highTFh cells were gated for expression of CXCR3, or were divided into four subsets based on the expression of CCR4, CCR6 and CXCR3: Th1 (CCR4negCCR6negCXCR3+), Th1Th17 (CCR4negCCR6+CXCR3+), CXCR3+Th17 (CCR4+CCR6+CXCR3+) and CXCR3+Th2 (CCR4+CCR6negCXCR3+).

## Slide 4
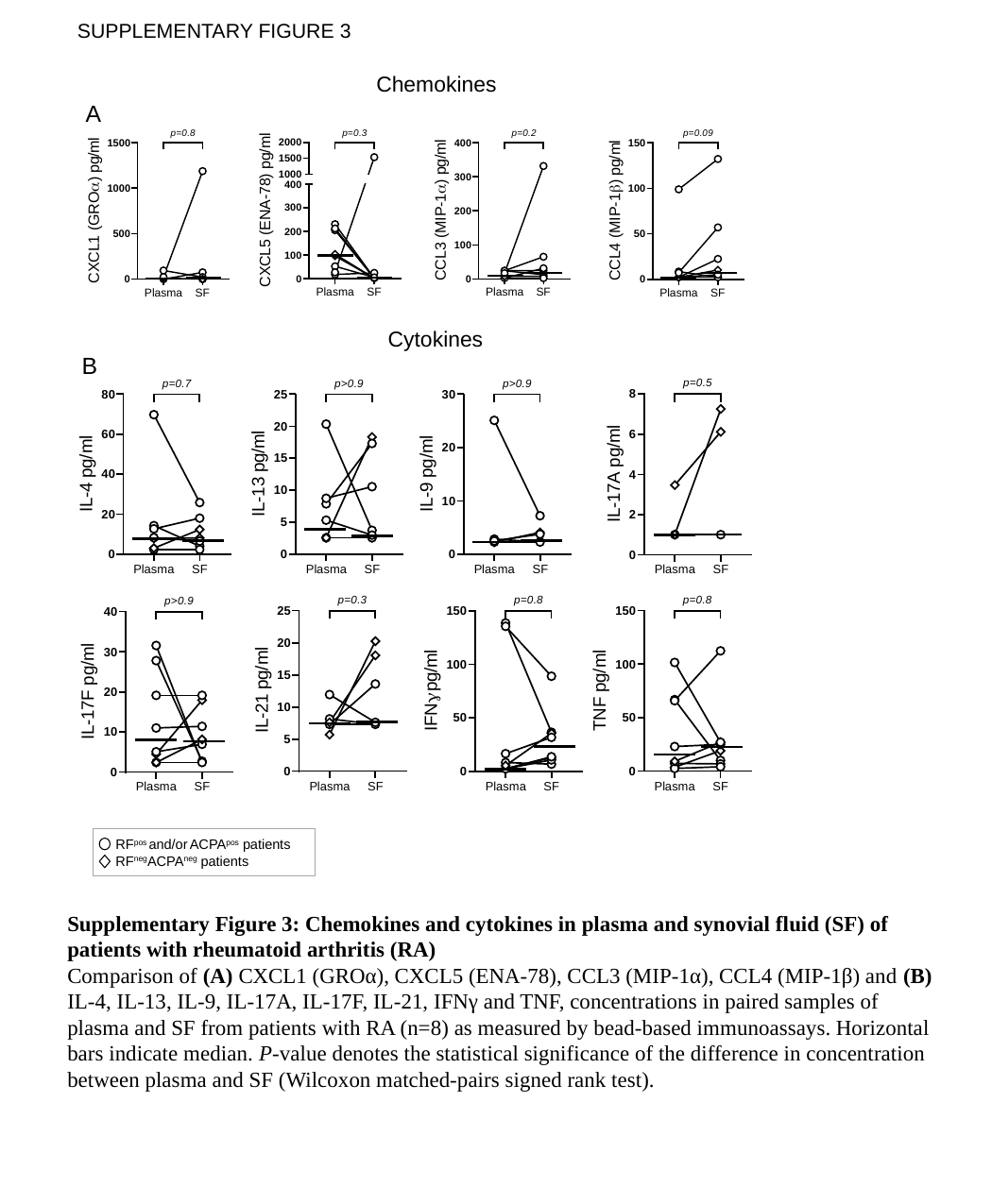

SUPPLEMENTARY FIGURE 3
Chemokines
A
Cytokines
B
RFpos and/or ACPApos patients
RFnegACPAneg patients
Supplementary Figure 3: Chemokines and cytokines in plasma and synovial fluid (SF) of patients with rheumatoid arthritis (RA)
Comparison of (A) CXCL1 (GROα), CXCL5 (ENA-78), CCL3 (MIP-1α), CCL4 (MIP-1β) and (B) IL-4, IL-13, IL-9, IL-17A, IL-17F, IL-21, IFNγ and TNF, concentrations in paired samples of plasma and SF from patients with RA (n=8) as measured by bead-based immunoassays. Horizontal bars indicate median. P-value denotes the statistical significance of the difference in concentration between plasma and SF (Wilcoxon matched-pairs signed rank test).

## Slide 5
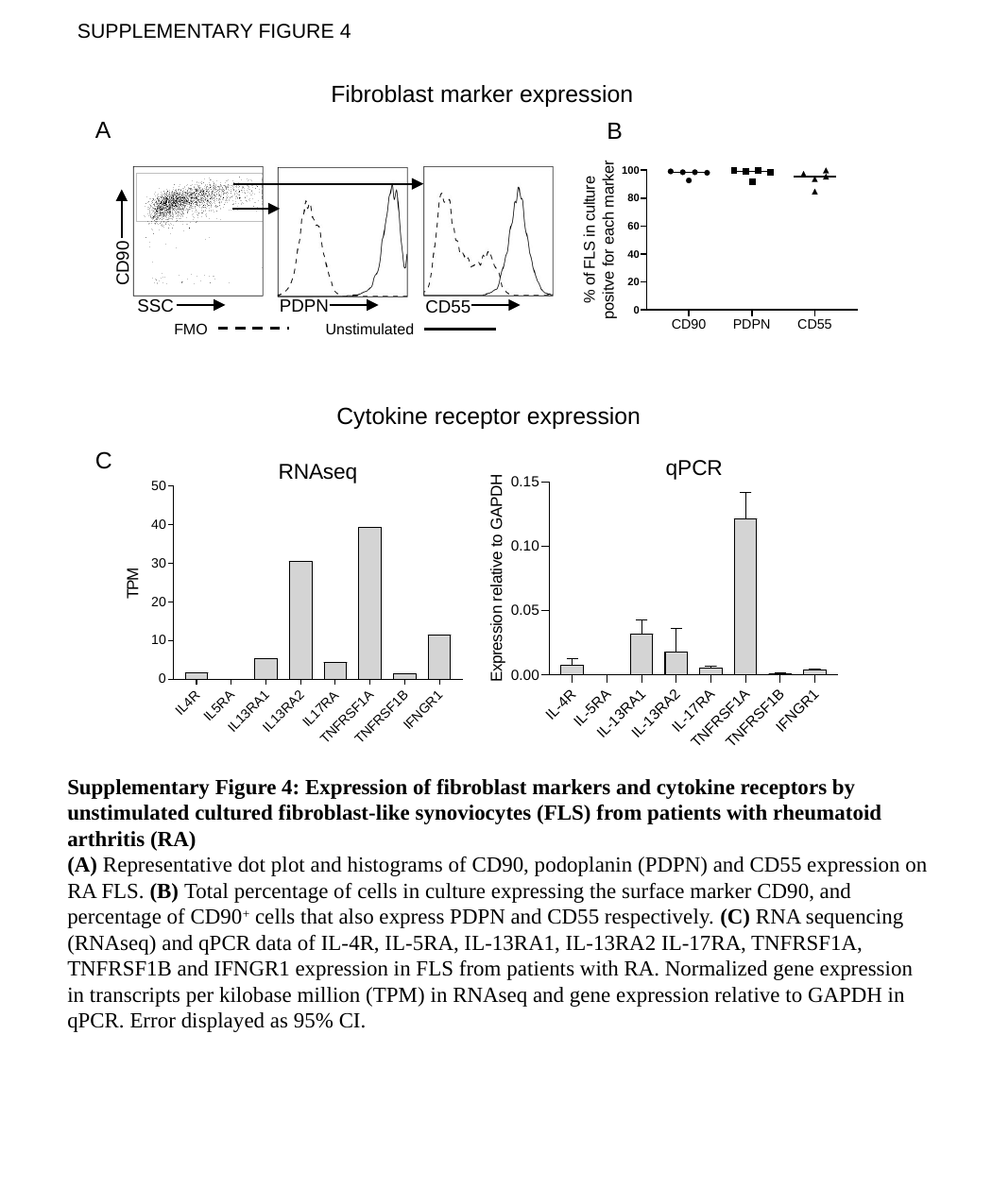

SUPPLEMENTARY FIGURE 4
Fibroblast marker expression
A
B
CD90
SSC
PDPN
CD55
Unstimulated
FMO
Cytokine receptor expression
C
Supplementary Figure 4: Expression of fibroblast markers and cytokine receptors by unstimulated cultured fibroblast-like synoviocytes (FLS) from patients with rheumatoid arthritis (RA)
(A) Representative dot plot and histograms of CD90, podoplanin (PDPN) and CD55 expression on RA FLS. (B) Total percentage of cells in culture expressing the surface marker CD90, and percentage of CD90+ cells that also express PDPN and CD55 respectively. (C) RNA sequencing (RNAseq) and qPCR data of IL-4R, IL-5RA, IL-13RA1, IL-13RA2 IL-17RA, TNFRSF1A, TNFRSF1B and IFNGR1 expression in FLS from patients with RA. Normalized gene expression in transcripts per kilobase million (TPM) in RNAseq and gene expression relative to GAPDH in qPCR. Error displayed as 95% CI.
